# Neurosphere Based Differentiation of Human iPSC Improves Astrocyte Differentiation

**DOI:** 10.1155/2016/4937689

**Published:** 2015-12-21

**Authors:** Shuling Zhou, Karolina Szczesna, Anna Ochalek, Julianna Kobolák, Eszter Varga, Csilla Nemes, Abinaya Chandrasekaran, Mikkel Rasmussen, Susanna Cirera, Poul Hyttel, András Dinnyés, Kristine K. Freude, Hasan X. Avci

**Affiliations:** ^1^Department of Veterinary Clinical and Animal Science, Faculty of Health and Medical Sciences, University of Copenhagen, 1870 Frederiksberg, Denmark; ^2^BioTalentum Ltd., Gödöllo 2100, Hungary; ^3^Molecular Animal Biotechnology Laboratory, SZIE, Gödöllo 2100, Hungary; ^4^Bioneer A/S, 2970 Hørsholm, Denmark

## Abstract

Neural progenitor cells (NPCs) derived from human induced pluripotent stem cells (iPSCs) are traditionally maintained and proliferated utilizing two-dimensional (2D) adherent monolayer culture systems. However, NPCs cultured using this system hardly reflect the intrinsic spatial development of brain tissue. In this study, we determined that culturing iPSC-derived NPCs as three-dimensional (3D) floating neurospheres resulted in increased expression of the neural progenitor cell (NPC) markers, *PAX6* and *NESTIN*. Expansion of NPCs in 3D culture methods also resulted in a more homogenous PAX6 expression when compared to 2D culture methods. Furthermore, the 3D propagation method for NPCs resulted in a significant higher expression of the astrocyte markers  *GFAP* and *aquaporin 4* (*AQP4*) in the differentiated cells. Thus, our 3D propagation method could constitute a useful tool to promote NPC homogeneity and also to increase the differentiation potential of iPSC towards astrocytes.

## 1. Introduction

Neural progenitor cells (NPCs) are self-renewing and multipotent cells, which are found in the embryonic, fetal, and adult mammalian central nervous system (CNS). These cells have the capacity to differentiate into neurons as well as glia cells [1, 2]. NPCs derived from pluripotent stem cells (PSCs), including embryonic stem cells (ESCs) and induced pluripotent stem cells (iPSCs), are an attractive* in vitro* model for studying the pathology of CNS disorders, for drug development and identification of novel drug targets [3, 4]. Compared with their* in vivo* counterparts NPCs are not as limited in regard to their proliferative status and differentiation capacity into various neural phenotypes* in vitro* [5–8].

NPCs are commonly isolated from different regions of murine and human brain by microdissection and subsequently cultured as free-floating aggregates using both classic and stirred suspension 3D culture system methods [9, 10]. It is common practice in the field of neuroscience and stem cell research to maintain and proliferate NPCs by using either two-dimensional (2D) adherent monolayer or three-dimensional (3D) floating neurosphere culture systems. Cells derived from the 3D culture system are thought to be more representative of the spatial cellular environment found in living organisms, including features of tissue-specific architecture, mechanical and biochemical cues, and cell-cell communication [11]. In accordance, neurospheres are widely accepted and used as* in vitro* assays to analyze the properties of NPCs [12]. This spatial integrity is not found in the 2D culturing system, which is considered to be the more artificial culturing technique [11].

A common approach of human NPCs derivation from iPSC involves neural induction by inhibition of SMAD signaling by means of two inhibitors (SB431542 and Noggin or LDN193189), followed by expansion of NPCs and subsequent terminal differentiation into neurons using the 2D culture system [8, 13, 14]. Yet, in order to model specific neurodegenerative diseases* in vitro* it is crucial that the culture methods display the desired regional and subtype specificity compared to the affected neurons of the patient. Consequently, disease modeling in 3D tissue culture systems has recently been successfully applied in Alzheimer's disease [15] and Parkinson's disease [16, 17] and to study glia cell differentiation [18, 19].

The human brain is made up of various subtypes of neurons but also by a substantial amount of glia cells (more than 50%) [20]. One subtype of glia cells is astrocytes, which play a complex and an essential role in neural maturation and homeostasis, including synaptic transmission and information processing by neural circuit functions [21]. Both neurons and glia cells, except for microglia, are derived from radial glia (RG) cells in the developing brain. RG cells are a NPC population, which originates from neuroepithelial cells the neural tube [5, 22]. During neurogenesis, 5/6 of RG cells divide asymmetrically into early bipolar intermediate progenitor (IP) cells which eventually differentiate into neurons. The remaining 1/6 of RG cells give rise to astrocyte and oligodendrocyte progenitor cells [23–25]. The differentiation from RG cells to early IP cells is accompanied by the loss of PAX6 expression [23]. Brain lipid-binding protein (BLBP) is a verified astrocyte progenitor marker, which was detected by following the expression pattern of brain BLBP in RG cells [20, 26]. Later, during development BLBP expression becomes restricted to astrocyte progenitors and downregulated in astrocytes [27]. One of the most commonly used astrocyte markers is glial fibrillary acidic protein (GFAP), which is expressed during CNS development and becomes restricted to astrocytes lineage [20]. Paired box 6 (PAX6) is an established NPC marker widely expressed in the radial glia cells and plays a crucial role in maintaining the NPC population, lineage-commitment, and gliogenesis [28–31].

Another aspect of neuronal differentiation, which may be a challenge under* in vitro* conditions, is the extended time frame (42–84 days) for achieving functional neuronal maturation [32, 33]. This can be accelerated by coculturing neurons with astrocytes. This makes astrocyte differentiation protocols highly desirable and needed for the neural maturation process [34, 35]. One of the main issues is that differentiation of astrocytes from fetal or adult postmortem CNS has been proven to be a difficult process with low efficiencies [36, 37]. Traditional 2D methods to generate sufficiently pure population of astrocytes derived from iPSCs and ESCs are on the other hand very time consuming (>180 days) [38]. Consequently, reliable 3D based differentiation methods, which can potentially enrich and accelerate astrocyte differentiation and maturation, would be beneficial in order to improve coculturing approaches.

In the present study, we describe a potentially efficient 3D method of astrocyte enrichment from human iPSC-derived NPCs. The method progresses through an initial phase of NPC formation with increasing expression of* PAX6* and* NESTIN*, which are NPC markers. Furthermore, we directly established a link between the expression of* BLBP*,* PAX6,* and astrocyte differentiation efficiency [27]. Our method provides an NPCs expansion protocol in 3D, which enriches the high PAX6 expression NPCs from mixed low and high PAX6 expression NPC pool and could be beneficial for astrocyte differentiation in which the efficiency can be monitored via* GFAP* and* AQP4* expression.

## 2. Materials and Methods

### 2.1. 2D Monolayer Culture of NPCs and Terminal Differentiation

The three human iPSC lines used in this study were generated by using the Sendai virus (CytoTune-iPS 2.0 Sendai Reprogramming Kit) (Life Technologies, Carlsbad, California, USA). Three cell lines were investigated in this study: clone N S5 and N S8 derived from a healthy 34-year-old male donor and clone RT S11 derived from a 33-year-old healthy female donor. NPCs cell lines were induced from each of these human iPSCs by dual inhibition of SMAD signaling with 10 *μ*M SB431542 (Sigma-Aldrich, St. Louis, Missouri, USA) and 100 ng/mL Noggin (R&D Systems, Minneapolis, Minnesota, USA). NPCs were expanded in neural maintenance medium and maintained on plates coated with poly-L-ornithine (Sigma-Aldrich) and laminin (Roche, Indianapolis, Indiana, USA) (POL/L). Terminal differentiation on POL/L coated plates was initiated using neural differentiation medium as described in the Media. Total time of terminal differentiation was 21 days.

### 2.2. 3D Neurospheres Culture of NPCs and Terminal Differentiation

Monolayer NPCs from all three lines (N S5, N S8, and RT S11) were dissociated into single cells with Accutase (Sigma-Aldrich). A total of 1 × 10^6^ cells/mL were plated on the low attachment dish (Sarstedt, Newton, Massachusetts, USA) in neural maintenance medium and maintained for 7 days as neurosphere cultures. Neurospheres were visualized in an inverted microscope under phase contrast mode. Three representative pictures of neurospheres were taken from 3 independent cultures with an inverted microscope under phase contrast mode (approximately 100 neurospheres were counted for each line, 4x magnification; OLYMPUS CKX41, Tokyo, Japan). Subsequently, the neurospheres were dissociated into single cells using Accutase and next seeded on plates coated with POL/L. Finally, the cells were differentiated for 21 days in neural differentiation medium.

### 2.3. Media

#### 2.3.1. Neural Maintenance Medium (NMM)

NMM consisted of a 50/50 mixture of DMEM/F-12 with GlutaMAX (Life Technologies) and neurobasal medium (Life Technologies). This basic media was supplemented with N2 (Life Technologies), B27 (Life Technologies), 2 mM L-Glutamine (Sigma-Aldrich), 10 ng/mL basic fibroblast growth factor (bFGF) (Life Technologies), 10 ng/mL epidermal growth factor (EGF) (Life Technologies), 1x nonessential amino acid (Sigma-Aldrich), and 50 U/mL penicillin/streptomycin (Life Technologies).

#### 2.3.2. Neural Differentiation Medium (NDM)

NDM was composed of a 50/50 mixture of DMEM/F-12 with GlutaMAX and neurobasal medium. This basic media was supplemented with N2, B27, 2 mM L-Glutamine, 1x nonessential amino acid, 0.2 mM ascorbic acid (Sigma-Aldrich), 25 *μ*M 2-mercaptoethanol (Life Technologies), and 50 U/mL penicillin/streptomycin.

### 2.4. Flow Cytometry

In order to perform flow cytometry analysis, NPCs harvested from 2D monolayer and 3D neurosphere were dissociated into single-cell suspension with Accutase. Dissociated cells were fixed with 4% paraformaldehyde (PFA) for 20 min at room temperature (RT) and subsequently permeabilized with 0,2% Triton X-100 for 20 minutes. Cells were stained for 1 hour at RT with* Alexa Fluor 647 mouse anti-NESTIN* and PE mouse anti-human Pax6 antibodies (BD Pharmingen, San Diego, California, USA). Flow cytometry analysis was performed using a “Flow Cytometer Cytomics FC 500” (Beckman Coulter, Pasadena, California, United States). A red solid state laser 635 nm and an argon laser 488 nm were used to detect NESTIN and PAX6 expression in NPCs. Subsequently, we set the specific gate to calculate the proportion of low PAX6 expressing NPCs from 2D and 3D culturing system. Data was analyzed using FlowJo software (version 7.6.5) from 3 independent cultures.

### 2.5. Immunocytochemistry (ICC), Imaging, and Quantification

To analyze neural markers cells were fixed in 4% PFA for 20 minutes at RT, washed in phosphate buffered saline (PBS), and permeabilized with 0.2% Triton X-100 diluted in PBS for 20 minutes. Afterwards, cells were blocked in 3% BSA + 0.2% Triton X-100 in PBS for 1 hour at RT. NPCs were incubated overnight at 4°C with the following primary antibodies: rabbit anti-PAX6 (Covance, Princeton, New Jersey, USA 1 : 250), mouse anti-NESTIN (Merck Millipore, Temecula, California, USA, 1 : 1000), rabbit anti-Musashi-1 (Merck Millipore, 1 : 400), mouse anti-SOX2 (R&D System, 1 : 100), and mouse anti-KI67 (Santa Cruz, Dallas, Texas, USA 1 : 800). Neurospheres were incubated overnight at 4°C with rabbit anti-PAX6 (Covance, 1 : 250) and mouse anti-NESTIN (Merck Millipore, 1 : 1000). Differentiated cells were incubated overnight at 4°C with mouse anti-MAP2 (Merck Millipore, 1 : 1000), rabbit anti-beta-III tubulin (Covance, 1 : 1000), mouse anti-beta-III tubulin (Santa Cruz, 1 : 1000), rabbit anti-AQP4 (Santa Cruz, 1 : 50), and rabbit anti-GFAP (DAKO, Glostrup, Denmark 1 : 1000). The primary antibodies were detected using the following fluorescence labeled secondary antibodies: Alexa Fluor 488 donkey anti-rabbit IgG (H + L), Alexa Fluor 594 donkey anti-mouse IgG (H + L), Alexa Fluor 488 donkey anti-mouse IgG (H + L), and Alexa Fluor 594 donkey anti-rabbit IgG (H + L) (all from Life Technologies). Finally, the cells were incubated for 20 min at RT with 4′,6-diamidino-2-phenylindole (DAPI) at a final concentration of 0.2 *μ*g/mL diluted in PBS to detect nuclei counterstaining. Unspecific binding of the secondary antibodies was excluded by using only secondary antibody controls. Cells were analyzed under fluorescent microscope equipped with 3D imaging module (AxioImager system with ApoTome, Carl Zeiss MicroImaging GmbH, and Jena, Germany) and controlled by AxioVision 4.8.1 Microscope software (Carl Zeiss MicroImaging GmbH, Jena, Germany). The number of proliferative cells (KI67^+^) was quantified. The numbers of neurons (TUBB3^+^) and astrocytes (GFAP^+^ and AQP4^+^) were quantified. Five pictures per coverslip were acquired from 3 independent cultures (20x magnification). On average, approximately 300 cells were counted for each picture.

### 2.6. Quantitative Real-Time Polymerase Chain Reaction (qPCR)

Total RNA was isolated from cultured cells using the RNeasyPlus Mini kit (Qiagen, Venlo, Limburg, Netherlands). Two micrograms of total RNA was transcribed to cDNA using the SuperScript VILO cDNA Synthesis kit (Life Technologies) in accordance with the manufacturer's instructions. QPCR reactions were performed using LightCycler 480 SYBR Green I (Roche) on a LightCycler 480 real-time PCR machine (Roche). In this study, the house-keeping gene* GAPDH* was used for data normalization. Samples were collected from 3 independent cultures. Data was analyzed based on 2^−ΔΔCt^ method [39]. All used primers for qPCR experiments are summarized in Table 1.

### 2.7. Statistics

Distribution of neurosphere diameter was tested using Kolmogorov-Smirnov normality test. A one-way analysis of variance (ANOVA) with a Tukey* post hoc* multiple comparison test was applied to compare the neurosphere diameter across all studied cell lines at days 4 and 7. Distribution of relative abundance of mRNA from the different genes was examined by using D'Agostino and Pearson omnibus normality test. Student's *t*-test was performed to compare the difference between groups. Pearson's correlation tests were performed to establish the correlation between the following: (1) the relative mRNA abundance of* PAX6* in 3D neurosphere NPCs and neurosphere diameter at day 7; (2) the relative mRNA abundance of* BLBP* and* PAX6* in NPCs and* GFAP* in differentiated cells over three studied cell lines. Distribution of proportion of KI67^+^, low PAX6 expression, TUBB3^+^, AQP4^+^, and GFAP^+^ cells was tested using Kolmogorov-Smirnov normality test. One-way ANOVA with a Tukey* post hoc* multiple comparison test and Student's *t*-test was performed to compare the difference between groups. A *p* value below 0.05 was considered significant. Results are reported as the Mean ± SEM (standard error of the mean). Data was analyzed using OriginPro Statistical software (version 9).

## 3. Results

### 3.1. Schematic Work Flow of 2D Monolayer Expansion, 3D Neurosphere Aggregation, and Terminal Differentiation of Human Neural Progenitor Cell Cultures (NPCs)

**Figure 1:**
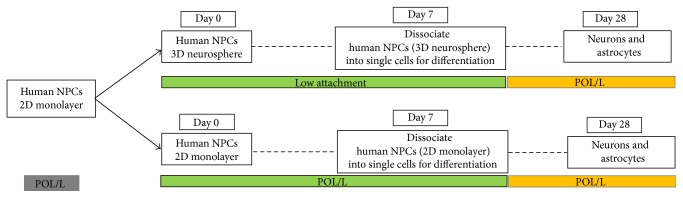
Schematic work flow of 2D monolayer expansion, 3D neurosphere aggregation, and terminal differentiation of human neural progenitor cell cultures (NPCs). POL/L (poly-L-ornithine/laminin coated plates); NPCs were dissociated into single cells and expanded in 2D monolayers or as 3D neurospheres. They were cultured in the NMM supplemented with growth factors bFGF and EGF for 7 days of expansion. Afterwards, NPCs from both culturing systems were dissociated into single cells and plated on POL/L coated plates for 21 days of terminal differentiation.

In order to investigate whether 3D culturing methods can enhance astrocyte differentiation from NPCs* in vitro*, we set up a direct comparison of 2D and 3D NPC expansion methods (Figure 1).

### 3.2. 3D Cultured NPCs Form Proliferating Neurospheres Positive for PAX6 and NESTIN

**Figure 2:**
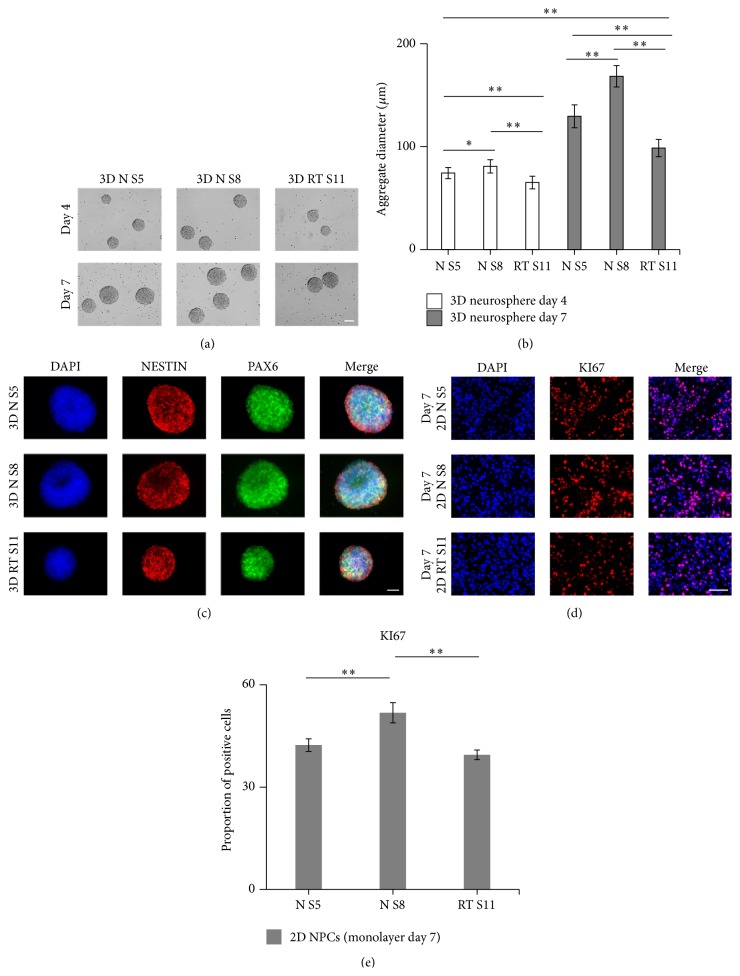
3D cultured NPCs form proliferating neurospheres positive for PAX6 and NESTIN. (a) Phase contrast images of neurospheres from all 3 lines (N S5, N S8, and RT S11) on day 4 and day 7. Scale bar = 100 *μ*m. (b) Analyses of neurosphere diameter on day 4 and day 7 of 3D suspension culture. Significant difference was tested via one-way ANOVA analysis with a Tukey* post hoc* multiple comparison test. Significant differences were indicated as follows: ^*∗*^ *p* < 0.05 and ^*∗∗*^ *p* < 0.01. Results were reported as Mean ± SEM of 9 fields from 3 independent cultures. (c) ICC staining of neurospheres (NESTIN, red, and PAX6, green) on day 7 from all 3 lines. Scale bar = 100 *μ*m. (d) Representative ICC of 2D NPCs (KI67). Scale bar = 100 *μ*m. (e) Quantification of KI67^+^ NPCs for 2D culturing systems. Results were reported as Mean ± SEM of 15 fields from 3 independent cultures, tested for significant differences (^*∗∗*^ *p* < 0.01) via one-way ANOVA analysis with a Tukey* post hoc* multiple comparison test.

We assessed the morphology of day 4 and 7 neurospheres by phase contrast imaging and their expression of early neural lineage markers (PAX6 and NESTIN) by immunocytochemistry. Neurosphere size was evaluated at days 4 and 7. We observed varying sizes of neurospheres within the different cell lines analyzed (Figure 2(a)). As shown in Figure 2(b), the diameter of neurospheres from NPC line N S8 (80.82 ± 6.43 *μ*m) was larger than N S5 (74.26 ± 5.45 *μ*m) and RT S11 (65.05 ± 6.18 *μ*m) at day 4. Even though we observed significant differences amongst cell lines, all of them were proliferative as neurospheres and increased in diameter during culturing. At day 7 the diameter of neurospheres was significantly increased in all lines (N S5 = 129.46 ± 11.11 *μ*m, N S8 = 168.35 ± 10.38 *μ*m, and RT S11 = 98.57 ± 8.37 *μ*m; *p* < 0.01) compared to day 4. One cell line (N S8) showed a significant larger diameter at day 7 compared to N S5 and RT S11 (*p* < 0.01). All lines analyzed (N S5, N S8, and RT S11) expressed NESTIN and PAX6 (Figure 2(c)), confirming NPC marker expression in the neurospheres. Additionally, to assess the proliferation capacity of NPCs expanded by the 2D method, we stained for the proliferation marker Ki-67. As shown in Figures 2(d) and 2(e), we observed significantly higher proportion of NPCs in N S8 which were positive for Ki-67 (KI67) in comparison to N S5 and RT S11 (*p* < 0.01). This result demonstrates that NPCs maintain the capacity to proliferate in 2D and 3D culturing system. Furthermore, 3D suspension culture induces the formation of neurospheres with the expression of NPC markers.

### 3.3. Comparison of NPC Markers Expression in 2D and 3D Cultures

**Figure 3:**
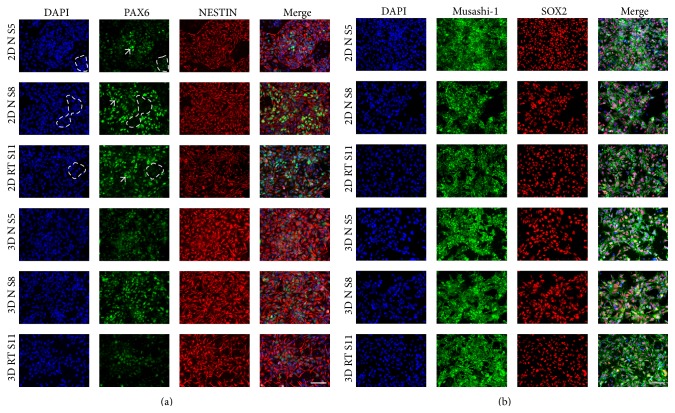
Comparison of NPC markers expression in 2D and 3D cultures. Both 2D and 3D NPCs were dissociated into single cells and plated onto POL/L coated coverslips. (a) Representative ICC of 2D NPCs and 3D NPCs (PAX6 and NESTIN). Scale bar = 100 *μ*m. (b) ICC of 2D NPCs and 3D NPCs (Musashi-1 and SOX2). Scale bar = 100 *μ*m.

ICC was performed to examine the expression level of NPC markers (PAX6, NESTIN, Musashi-1, and SOX2) in 2D and 3D NPCs. Both were dissociated and plated as single cells on day 7. As expected, 2D and 3D NPCs were positive for NESTIN and PAX6 expression (Figure 3(a)). Furthermore ICC was performed using SRY- (sex determining region Y-) box 2 (SOX2) and Musashi RNA-binding protein 1 (Musashi-1), which label self-renewing NPCs in the nucleus (SOX2) and cytoplasm (Musashi-1) (Figure 3(b)). Interestingly, PAX6 expression was heterogeneous in 2D NPC cultures. Some areas showed low PAX6 expression (encircled with dashed line), whilst other areas showed high PAX6 expression (arrows) (Figure 3(a)). In contrast the expression pattern of PAX6 in 3D dissociated and plated NPCs was more uniformly distributed (Figure 3(a)). Our results show that NPCs cultured in both 2D and 3D culture system express NPC markers.

### 3.4. NPCs in 3D Neurospheres Showed Increased Homogenous PAX6 Expression

**Figure 4:**
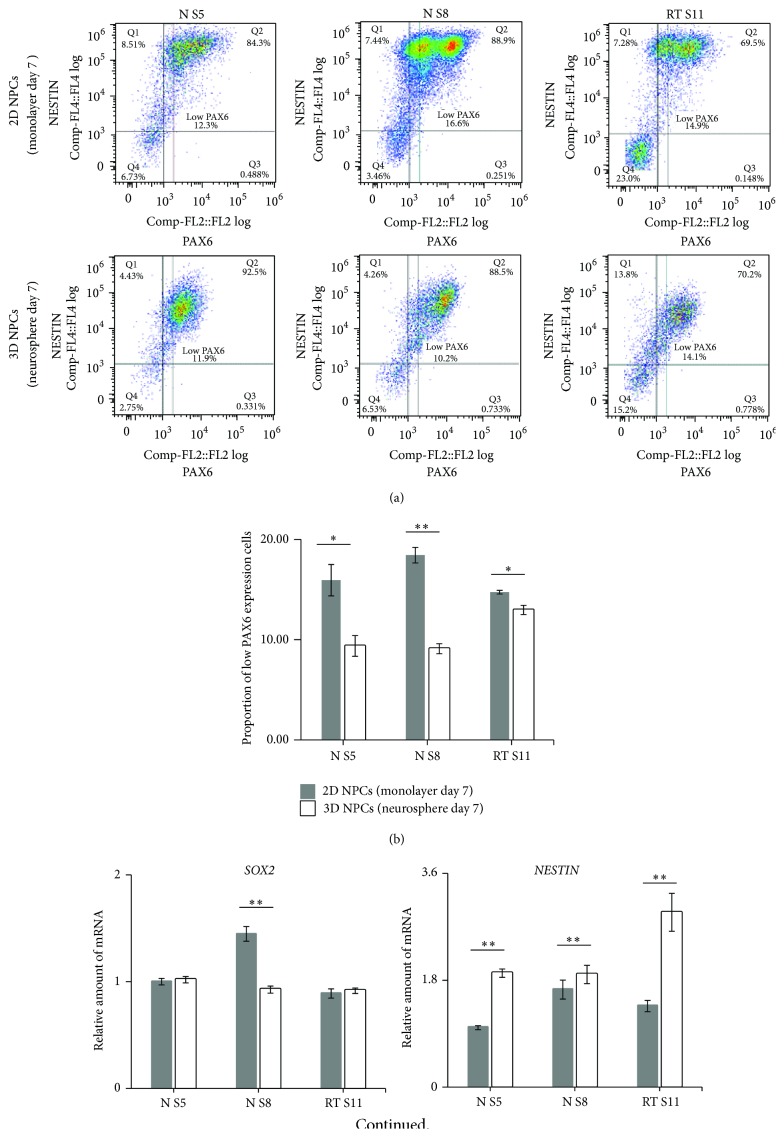
NPCs in 3D neurospheres have increased homogenous PAX6 expression. (a) Flow cytometer dot plot demonstrated that PAX6 expression in 3D NPCs was more homogeneous compared to 2D NPCs. (b) Specific gating was applied to quantify the low PAX6 expression population in 2D NPCs and 3D NPCs. Student's *t*-test (^*∗*^ *p* < 0.05 and ^*∗∗*^ *p* < 0.01). The bars represent the Mean ± SEM of 3 independent cultures set. (c) QPCR analysis of* SOX2*,* NESTIN*, and* PAX6* expression of cultured 2D NPCs and 3D NPCs. Expression values were normalized to* GAPDH* (reference gene). Subsequently, the expression values were calculated as relative amount of mRNA versus expression values of N S5 (monolayer) which was set to 1. Test for significant difference (^*∗∗*^ *p* < 0.01) using Student's *t*-test. The bars represent the Mean ± SEM of 3 independent cultures set. (d) A scatter plot and trend line (Pearson correlation) showed correlation between day 7 neurosphere diameter and the average relative amount of mRNA of* PAX6* in 3D NPCs.

Flow cytometry was implemented to study the expression level of PAX6 and NESTIN in 2D and 3D NPCs. The intensity of the PAX6 signal in the FACS analyses revealed two apparently divergent populations in 2D NPCs whilst 3D NPCs showed only one distinct population. The appearance of two individual populations was most prominent with the 2D NS8 cells (Figure 4(a)). Subsequently, specific gating was applied to compare the proportion of low PAX6 expression NPCs in 2D and 3D culturing system. Our results presented in Figure 4(b) show significant decrease of low PAX6 expression NPCs in 3D culturing system. We further analyzed all cell lines used for the two culture systems by qPCR to investigate the expression of NPC markers (*SOX2*,* PAX6,* and* NESTIN*). The abundance of* PAX6* and* NESTIN* was significantly increased (*p* < 0.01) in 3D cultured NPCs in comparison with 2D cultured NPCs in all investigated cell lines (Figure 4(c)). However, we were not able to detect similar expression tendencies for* SOX2* using the 3D culture system. Pearson's correlation was used to evaluate the relationship between the NPC marker* PAX6* and neurosphere diameter. Day 7 neurosphere diameter had strongly positive (Pearson's *r* = 0.68, *p* = 0.043) correlation with mRNA abundance of* PAX6* in 3D cultured NPCs (Figure 4(d)). This indicates a direct correlation between* PAX6* expression and neurosphere diameter. Taken together, we observe that within the 3D culturing system NPCs displaying low PAX6 expression become eliminated and subsequently NPC marker expression (*PAX6* and* NESTIN*) is increased.

### 3.5. Neurosphere Aggregation Promotes Astrocyte Differentiation

**Figure 5:**
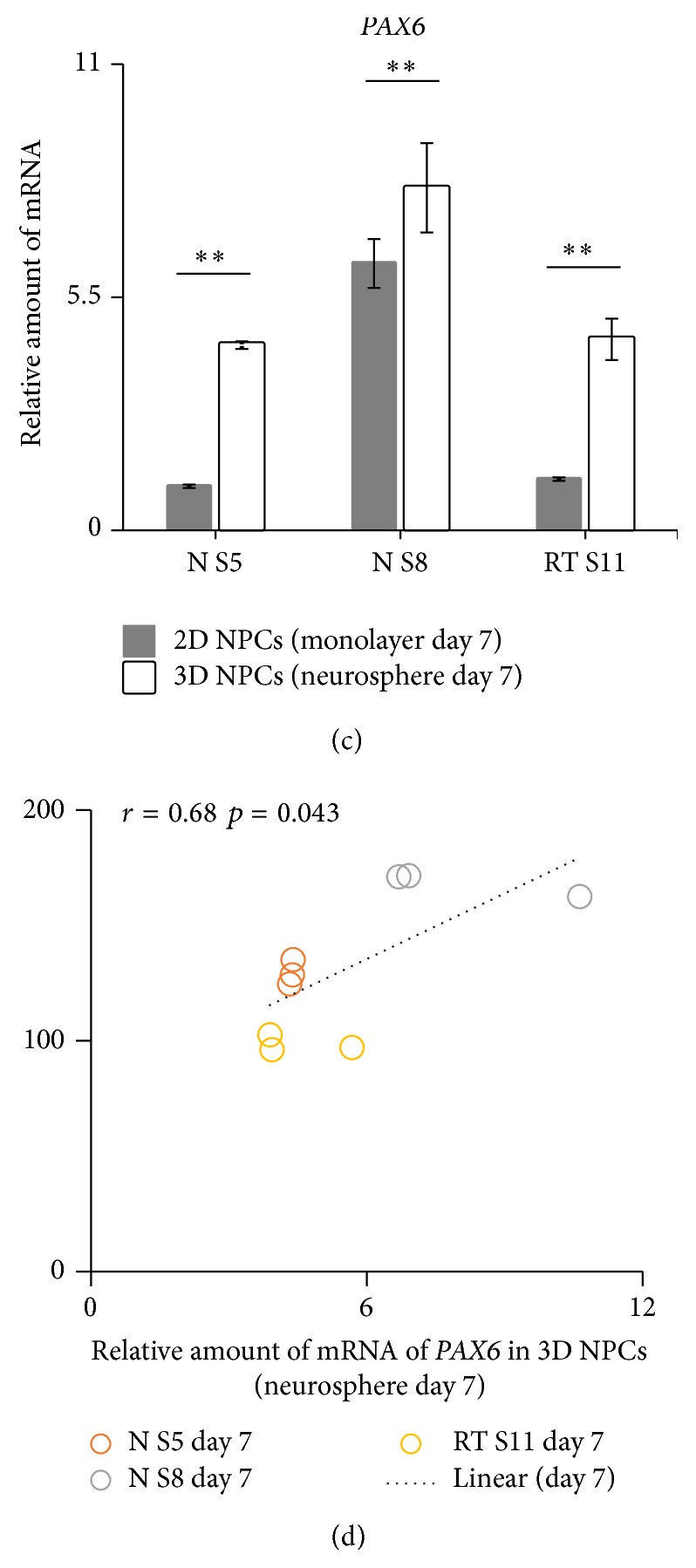
Neurosphere aggregation promotes astrocyte differentiation. (a) ICC of 21-day differentiated neurons and astrocytes (TUBB3 and GFAP) derived from 2D and 3D NPCs. (b) ICC of 21-day neurons (MAP2 and TUBB3) differentiated from 2D and 3D NPCs. (c) Quantification of TUBB3^+^ and GFAP^+^ cells from iPSC-derived NPCs. Results were reported as Mean ± SEM of 15 fields from 3 independent cultures, tested for significant difference (^*∗*^ *p* < 0.05 and ^*∗∗*^ *p* < 0.01) using Student's *t*-test. (d) Gene expression by qPCR analysis of* TUBB3*,* MAP2,* and* GFAP*. The expression values were normalized to* GAPDH*. Subsequently, the expression values were calculated as relative amount of mRNA versus expression values of N S5 (derived from 2D NPCs) 21-day differentiated cells, which was set to 1. Data was reported as Mean ± SEM of 3 independent cultures, tested for significant difference (^*∗*^ *p* < 0.05 and ^*∗∗*^ *p* < 0.01) using Student's *t*-test. (e) A scatter plot and trend line (Pearson correlation) showed correlation between the average relative amount of* GFAP* (21-day differentiated cells) and* BLBP* (NPCs) mRNA. The expression values of* BLBP* in NPCs were calculated as relative amount of mRNA versus expression values of N S5 (monolayer) which was set to 1. Data was obtained from 3 independent cultures. (f) A scatter plot and trend line (Pearson correlation) showed correlation between the average relative amount of* GFAP* (21-day differentiated cells) and* PAX6* (NPCs) mRNA.

**Figure 6:**
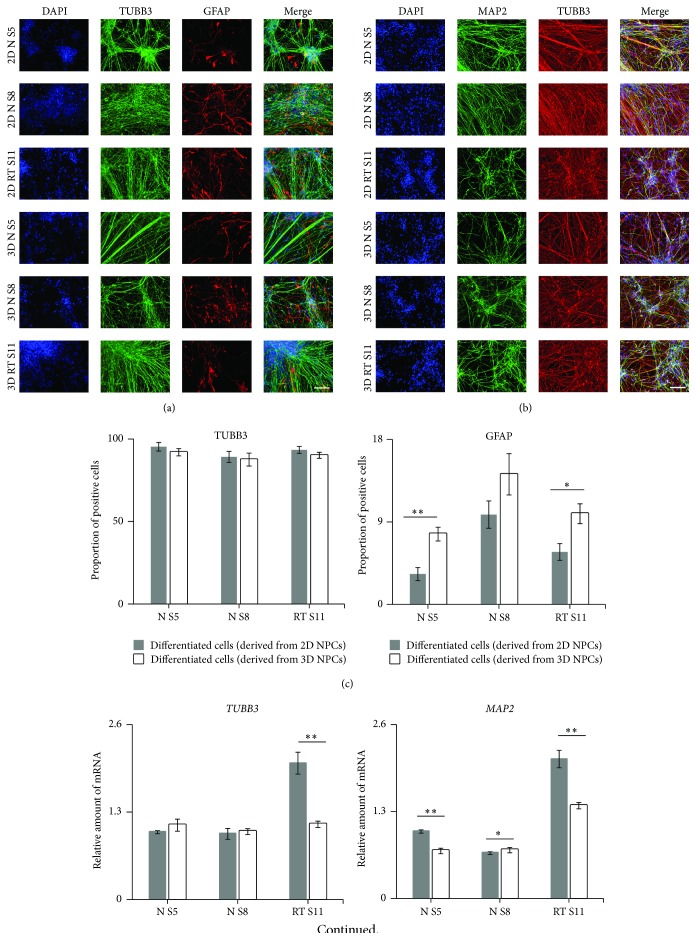
Increased astrocyte differentiation mediated by neurospheres. (a) ICC of 21-day differentiated astrocytes (AQP4) derived from 2D and 3D NPCs. (b) Quantification of AQP4^+^ positive astrocytes derived from 2D and 3D NPCs. Results were reported as Mean ± SEM of 15 fields from 3 independent cultures (^*∗∗*^ *p* < 0.01), using Student's *t*-test. (c) Analysis of gene expression by qPCR of* AQP4* and* TBR2*. The expression values were normalized to* GAPDH* gene expression. Subsequently, the expression values were calculated as relative amount of mRNA versus expression values of N S5 (derived from 2D NPCs) 21-day differentiated cells, which was set to 1. Data was reported as Mean ± SEM of 3 independent cultures, tested for significant difference (^*∗∗*^ *p* < 0.01) using Student's *t*-test.

The 2D and 3D NPCs were dissociated and differentiated on POL/L plates for 21 days. ICC analysis showed that the majority of cells derived from NPCs cultured in the two different systems were positive for neuronal markers (tubulin, beta 3 class III (TUBB3), and microtubule-associated protein 2 (MAP2); Figures 5(a) and 5(b)). A subset of cells was positive for GFAP expression, specific for astrocytes, and showed that the proportion of GFAP positive cells was higher in NPCs derived from 3D versus 2D cultures, primarily observed in N S5 and RT S11 lines (*p* < 0.01) (Figure 5(c)). We also evaluated the abundance of neuronal and astrocyte marker expression via qPCR in cells derived from 2D and 3D NPCs, which have been differentiated for 21 days. At this time point a subset of 3D derived cells expressed higher levels of* GFAP* compared to 2D derived cells (Figure 5(d)). Subsequently, to confirm that 3D culture system promotes astrocyte differentiation we evaluate the expression of another astrocyte marker aquaporin 4 (AQP4). As shown in Figures 6(a) and 6(b), significant higher proportion of AQP4 positive cells were observed in cells differentiated from 3D cultured NPCs (*p* < 0.01). Furthermore, qPCR was performed to verify the ICC quantification results. Significantly higher expression of* AQP4* was observed in cells differentiated from 3D culturing method (Figure 6(c)) (*p* < 0.01). Together these data indicate that neurosphere aggregation could promote astrocyte differentiation. Similar expression patterns of* MAP2* and* TUBB3* and the population of TUBB3 positive neurons were detected in neurons derived from NPCs using both culture systems (Figures 5(c) and 5(d)). This implies that there is no apparent difference in neuronal maturation between the two methods. The correlation between the relative expression abundance of* BLBP* and* PAX6* in NPCs and* GFAP* in 21-day differentiated cells was evaluated using the Pearson correlation analysis (Figures 5(e) and 5(f)). Pearson correlation analysis demonstrated that the expression of astrocyte progenitor marker* BLBP *and NPC marker* PAX6* during NPCs stage has a significant positive correlation (*r* = 0.738, *p* = 4.59 × 10^−4^ and *r* = 0.703, *p* = 1.12 × 10^−3^) to the expression of astrocyte marker (*GFAP*) in the astrocytes derived from all studied NPCs lines. Therefore,* BLBP* and* PAX6* expression during NPC stages can be used as an indicator for potential differentiation into the astrocyte lineage as verified by* GFAP* expression. Furthermore, we performed qPCR analysis to prove that the increase of PAX6 expression and the promotion of astrocyte differentiation were due to the loss of cells with low PAX6 expression during neurosphere formation. These low PAX6 expressing cells are considered early IP cells. Our qPCR results demonstrated a significant decrease of expression of the IP cell marker eomesodermin (*TBR2*) in cells differentiated from 3D cultured NPCs compared to 2D cultured NPCs (Figure 6(c)) (*p* < 0.01). Herein it can be concluded that the 3D culture method might promote the astrocytes differentiation and provoke an inhibition of the IP cells generation.

## 4. Discussion

The present study describes the fate and cellular properties of human NPCs derived from human iPSC and cultured as monolayers (2D) and neurospheres (3D). Human NPCs derived from iPSCs have traditionally been maintained and proliferated in 2D culture system, demonstrating an efficient differentiation into the neuronal lineage [13, 14]. However, the 2D culture systems are poor in recapitulating the spatially well-organized intercellular relationships characteristic of neural* in vivo* development in the CNS. In contrast, the neurospheres generated from 3D free-floating aggregates of NPCs with a certain spatial degree of complexity better mimic the main features of brain tissue and can therefore be considered as more relevant* in vitro* models [46]. Thus, neurospheres as a mean to produce neurons and astrocytes could provide a reliable* in vitro* cellular model for understanding CNS disorders and can play a relevant role in cell-based drug screening [11, 15, 17]. Previously, other groups have described the generation of neurospheres from brain-derived NPCs [47, 48]. We have employed the same method to generate neurospheres from iPSC-derived NPCs making the methodology much more versatile for implementation for* in vitro* cell modeling. Our findings show that the neurosphere diameter increases from day 4 to day 7, which indicates the ability of cell proliferation within the 3D culture system. Further, ICC characterization showed a very uniform expression of NPC markers in neurospheres, such as PAX6 and NESTIN. We also monitored the proliferation capacity of 2D NPCs via ICC for KI67 and observed a higher proportion of KI67 positive cells in N S8 compared to N S5 and RT S11. The proliferative capacity of 2D NPCs is directly correlated to the size of the neurospheres in the 3D system. Therefore, highly proliferative lines in the 2D system generate larger neurospheres in the 3D system. It might be that these neurospheres are proliferative NPCs, similar to brain-derived NPCs.

In the present study, we further compared the NPC identity and differentiation ability of NPCs cultured in 2D and 3D culturing systems. We chose PAX6, NESTIN, SOX2, and Musashi-1 (NPC markers) to evaluate the neural identity of our 2D and 3D NPCs. PAX6 is uniformly expressed in early neural ectoderm cells of human fetuses and in NPCs differentiated from human ESCs [49]. Pan-neuronal markers (SOX2, NESTIN, and Musashi-1) are widely expressed in all NPCs [50–53]. Some groups also found that PAX6, NESTIN, and SOX2 are expressed in iPSC-derived NPCs [8, 13, 54]. Moreover, PAX6, NESTIN, SOX2, and Musashi-1 are also the RG cell markers [55, 56]. Our results demonstrated that both 2D and 3D NPCs expressed these NPC markers, but* PAX6* and* NESTIN* expression levels were significantly higher in 3D NPCs. This indicates that neurosphere aggregation promotes* PAX6* and* NESTIN* expression. Furthermore, a more homogenous PAX6 expression was detected in 3D NPC populations suggesting that an uneven distributed PAX6 expression pattern in 2D NPCs was avoided by neurospheres aggregation. PAX6 is an established NPC marker expressed in RG cells which plays an essential role in maintaining the NPC population, neuronal and glial differentiation [29]. Previous study has shown that neurospheres are largely composed of RG cells (high PAX6 expression) other than early IP cells (low PAX6 expression) [57]. As a consequence NPC with low expression levels of PAX6 cannot form neurospheres. Consistent with this study, our results indicate that we could enrich the high PAX6 expression cells from mixed low and high PAX6 expression NPCs pool by using 3D culturing method. Furthermore, expression of* PAX6* induces production of larger neurospheres [58]. Consistent with this study, our results showed that expression of* PAX6* in 3D NPCs was positively correlated to subsequent neurosphere diameters at day 7.

Herein, we demonstrated that human iPSC-derived NPCs cultured under 2D and 3D conditions differentiated into cells positive for mature neuronal markers (MAP2 and TUBB3) and the astrocyte markers (GFAP and AQP4). The overall process of directing differentiation of human iPSCs-derived NPCs to neurons and astrocytes* in vitro* has been described earlier [59]. As a novel finding, our experiments demonstrated that propagation under the 3D conditions resulted in a significantly higher yield of cells with* GFAP* and* AQP4* expression compared to the 2D condition. Hence, we draw a conclusion that 3D NPCs culturing methods stimulate astrocyte differentiation. This observation is in agreement with previously described findings, which show efficient 3D differentiation of ESC-derived NPCs to glial cells of the peripheral nervous system, in particular Schwann cells [18]. Moreover, we demonstrated that the* GFAP* expression in astrocytes was significantly positively correlated to the astrocyte progenitor marker* BLBP* expression in NPCs, which is in accordance with observations of other groups [20, 26, 27]. Furthermore, it has been shown that abolishing the expression of PAX6 leads to the downregulation of* GFAP* expression, resulting in inhibition of astrocyte maturation [30, 60]. Similar to these studies, we found that the* GFAP* expression in astrocytes was strongly and positively associated with* PAX6* expression pattern during the NPC stage. These correlation studies underline the importance of* BLBP* and* PAX6* expression in NPCs for differentiation towards an astrocyte fate.

Single-cell mRNA profiling indicates that neurospheres are composed of RG cells (high PAX6 expression) other than early IP cells or IP cells (low PAX6 expression) [57]. Similar to this study, we observed that there was a significant decrease of expression of the IP cell marker* TBR2* in the differentiated cells derived from 3D NPCs when compared to 2D NPCs. This indicated that neurosphere formation is associated with the loss of early IP cells (low PAX6 expression cells), resulting in the decrease of* TBR2* expression in differentiated cells. During neurogenesis, IP cells asymmetrically divide into neurons and IP daughter cells [25]. Moreover, 1/6 of RG cells (high PAX6 expression cells) proceed to produce astrocyte and oligodendrocyte progenitors, resulting in the generation of astrocytes and oligodendrocytes [24, 25]. However, we do not observe the suppression of neuronal differentiation in our 2D analyzed cell lines. The reason for this may be due to the fact that only a small population of RG cells would proceed to give rise to glia cells; the remaining RG cells are still able to differentiate into neurons. Taken together, our results demonstrate that astrocyte differentiation is promoted by 3D propagation and relies on the increase of neurosphere aggregation.

## 5. Conclusion

In summary, we established a reliable NPC culture system in 3D that efficiently enriches GFAP and AQP4 positive astrocytes during terminal differentiation. Propagation of NPCs using the 3D culture system promotes the expression of RG cell markers (*PAX6* and* NESTIN*). Subsequently,* GFAP* and* AQP4* expression is increased during terminal astrocyte differentiation in the cells differentiated from 3D cultured NPCs. These results revealed an attractive method to improve the astrocyte differentiation ability from iPSC-derived NPCs in a shorter time frame and with higher efficiency compared to derivations from 2D cultured NPCs.

## Figures and Tables

**Table 1:** Human specific primers used for qPCR analysis.

Symbol	Sequence 5′-3′	Product size (BP)	Gene name	Reference number (NCBI)	Reference
*GAPDH*	GGTCACCAGGGCTGCTTTTA GGATCTCGCTCCTGGAAGATG	195	Glyceraldehyde-3-phosphate dehydrogenase	NM_001289746.1	[40]

*NESTIN*	CTCCAGAAACTCAAGCACC TCCTGATTCTCCTCTTCCA	145	Nestin	NM_006617.1	[41]

*SOX2*	TTCACATGTCCCAGCACTACCAGA TCACATGTGTGAGAGGGGCAGTGTGC	80	SRY- (sex determining region Y-) box 2	NM_003106.3	[42]

*PAX6*	TGGTATTCTCTCCCCCTCCT TAAGGATGTTGAACGGGCAG	126	Paired box 6	XM_005252958.3	[43]

*BLBP*	GGCTTTGCCACTAGGCAGG TGACCACTTTGTCTCCTTCTTGA	76	Brain lipid-binding protein	XM_005266858.2	[44]

*TUBB3*	TCCGCTCAGGGGCCTTTGGAC GCTCCGCCCCCTCCGTGTAG	108	Tubulin, beta 3 class III	NM_001197181.1	

*TBR2*	CACCGCCACCAAACTGAGAT CGAACACATTGTAGTGGGCAG	109	Eomesodermin	NM_001278182.1	[45]

*AQP4*	AGCAGTCACAGCGGAATTTCT TCTGTTCCACCCCAGTTGATG	81	Aquaporin 4	XM_011525942.1	[44]

*MAP2*	AGTTCAGGCCCACTCTCCCTCC GGGAGCCAGAGCTGATTCCCCA	127	Microtubule-associated protein 2	XM_011511198.1	

*GFAP*	AGGTCCATGTGGAGCTTGAC GCCATTGCCTCATACTGCGT	82	Glial fibrillary acidic protein	NM_001242376.1	[44]

## References

[1] Gage F. H. (2000). Mammalian neural stem cells. *Science*.

[2] Breunig J. J., Haydar T. F., Rakic P. (2011). Neural stem cells: historical perspective and future prospects. *Neuron*.

[3] Thomson J. A., Itskovitz-Eldor J., S.S. Shapiro, (1998). Embryonic stem cell lines derived from human blastocysts. *Science*.

[4] Takahashi K., Tanabe K., Ohnuki M. (2007). Induction of pluripotent stem cells from adult human fibroblasts by defined factors. *Cell*.

[5] Conti L., Cattaneo E. (2010). Neural stem cell systems: physiological players or in vitro entities?. *Nature Reviews Neuroscience*.

[6] Schwartz P. H., Brick D. J., Stover A. E., Loring J. F., Müller F.-J. (2008). Differentiation of neural lineage cells from human pluripotent stem cells. *Methods*.

[7] Broccoli V., Giannelli S. G., Mazzara P. G. (2014). Modeling physiological and pathological human neurogenesis in the dish. *Frontiers in Neuroscience*.

[8] Boissart C., Poulet A., Georges P. (2013). Differentiation from human pluripotent stem cells of cortical neurons of the superficial layers amenable to psychiatric disease modeling and high-throughput drug screening. *Translational Psychiatry*.

[9] Ahmed S. (2009). The culture of neural stem cells. *Journal of Cellular Biochemistry*.

[10] Rodrigues C. A. V., Fernandes T. G., Diogo M. M., da Silva C. L., Cabral J. M. S. (2011). Stem cell cultivation in bioreactors. *Biotechnology Advances*.

[11] Pampaloni F., Reynaud E. G., Stelzer E. H. K. (2007). The third dimension bridges the gap between cell culture and live tissue. *Nature Reviews Molecular Cell Biology*.

[12] Marshall G. P., Reynolds B. A., Laywell E. D. (2007). Using the neurosphere assay to quantify neural stem cells in vivo. *Current Pharmaceutical Biotechnology*.

[13] Chambers S. M., Fasano C. A., Papapetrou E. P., Tomishima M., Sadelain M., Studer L. (2009). Highly efficient neural conversion of human ES and iPS cells by dual inhibition of SMAD signaling. *Nature Biotechnology*.

[14] Shi Y., Kirwan P., Smith J., Robinson H. P. C., Livesey F. J. (2012). Human cerebral cortex development from pluripotent stem cells to functional excitatory synapses. *Nature Neuroscience*.

[15] Choi Y. J., Park J., Lee S.-H. (2013). Size-controllable networked neurospheres as a 3D neuronal tissue model for Alzheimer's disease studies. *Biomaterials*.

[16] Brito C., Simao D., Costa I. (2012). Generation and genetic modification of 3D cultures of human dopaminergic neurons derived from neural progenitor cells. *Methods*.

[17] Simão D., Pinto C., Piersanti S. (2015). Modeling human neural functionality *in vitro*: 3D culture for dopaminergic differentiation. *Tissue Engineering Part A*.

[18] Ziegler L., Grigoryan S., Yang I. H., Thakor N. V., Goldstein R. S. (2011). Efficient generation of schwann cells from human embryonic stem cell-derived neurospheres. *Stem Cell Reviews and Reports*.

[19] Machon O., Backman M., Krauss S., Kozmik Z. (2005). The cellular fate of cortical progenitors is not maintained in neurosphere cultures. *Molecular and Cellular Neuroscience*.

[20] Rowitch D. H., Kriegstein A. R. (2010). Developmental genetics of vertebrate glial-cell specification. *Nature*.

[21] Sofroniew M. V., Vinters H. V. (2010). Astrocytes: biology and pathology. *Acta Neuropathologica*.

[22] Nat R., Nilbratt M., Narkilahti S., Winblad B., Hovatta O., Nordberg A. (2007). Neurogenic neuroepithelial and radial glial cells generated from six human embryonic stem cell lines in serum-free suspension and adherent cultures. *Glia*.

[23] Lui J. H., Hansen D. V., Kriegstein A. R. (2011). Development and evolution of the human neocortex. *Cell*.

[24] DeAzevedo L. C., Fallet C., Moura-Neto V., Daumas-Duport C., Hedin-Pereira C., Lent R. (2003). Cortical radial glial cells in human fetuses: depth-correlated transformation into astrocytes. *Journal of Neurobiology*.

[25] Gao P., Postiglione M. P., Krieger T. G. (2014). Deterministic progenitor behavior and unitary production of neurons in the neocortex. *Cell*.

[26] Anthony T. E., Klein C., Fishell G., Heintz N. (2004). Radial glia serve as neuronal progenitors in all regions of the central nervous system. *Neuron*.

[27] Barry D., McDermott H. (2005). Differentiation of radial glia from radial precursor cells and transformation into astrocytes in the developing rat spinal cord. *Glia*.

[28] Hochstim C., Deneen B., Lukaszewicz A., Zhou Q., Anderson D. J. (2008). Identification of positionally distinct astrocyte subtypes whose identities are specified by a homeodomain code. *Cell*.

[29] Osumi N., Shinohara H., Numayama-Tsuruta K., Maekawa M. (2008). Concise review: Pax6 transcription factor contributes to both embryonic and adult neurogenesis as a multifunctional regulator. *Stem Cells*.

[30] Sakurai K., Osumi N. (2008). The neurogenesis-controlling factor, Pax6, inhibits proliferation and promotes maturation in murine astrocytes. *The Journal of Neuroscience*.

[31] Englund C., Fink A., Lau C. (2005). Pax6, Tbr2, and Tbr1 are expressed sequentially by radial glia, intermediate progenitor cells, and postmitotic neurons in developing neocortex. *The Journal of Neuroscience*.

[32] Ricciardi S., Ungaro F., Hambrock M. (2012). CDKL5 ensures excitatory synapse stability by reinforcing NGL-1-PSD95 interaction in the postsynaptic compartment and is impaired in patient iPSC-derived neurons. *Nature Cell Biology*.

[33] Verpelli C., Carlessi L., Bechi G. (2013). Comparative neuronal differentiation of self-renewing neural progenitor cell lines obtained from human induced pluripotent stem cells. *Frontiers in Cellular Neuroscience*.

[34] Tang X., Zhou L., Wagner A. M. (2013). Astroglial cells regulate the developmental timeline of human neurons differentiated from induced pluripotent stem cells. *Stem Cell Research*.

[35] Zhang Y., Pak C., Han Y. (2013). Rapid single-step induction of functional neurons from human pluripotent stem cells. *Neuron*.

[36] Haidet-Phillips A. M., Hester M. E., Miranda C. J. (2011). Astrocytes from familial and sporadic ALS patients are toxic to motor neurons. *Nature Biotechnology*.

[37] Verwer R. W. H., Sluiter A. A., Balesar R. A. (2007). Mature astrocytes in the adult human neocortex express the early neuronal marker doublecortin. *Brain*.

[38] Krencik R., Weick J. P., Liu Y., Zhang Z.-J., Zhang S.-C. (2011). Specification of transplantable astroglial subtypes from human pluripotent stem cells. *Nature Biotechnology*.

[39] Livak K. J., Schmittgen T. D. (2001). Analysis of relative gene expression data using real-time quantitative PCR and the 2^−ΔΔ*C*_T_^ method. *Methods*.

[40] Li H., Bian C., Liao L., Li J., Zhao R. C. (2011). miR-17-5p promotes human breast cancer cell migration and invasion through suppression of HBP1. *Breast Cancer Research and Treatment*.

[41] Nemati S., Hatami M., Kiani S. (2011). Long-term self-renewable feeder-free human induced pluripotent stem cell–derived neural progenitors. *Stem Cells and Development*.

[42] Okita K., Matsumura Y., Sato Y. (2011). A more efficient method to generate integration-free human iPS cells. *Nature Methods*.

[43] Kirkeby A., Grealish S., Wolf D. A. (2012). Generation of regionally specified neural progenitors and functional neurons from human embryonic stem cells under defined conditions. *Cell Reports*.

[44] Spandidos A., Wang X., Wang H., Seed B. (2009). PrimerBank: a resource of human and mouse PCR primer pairs for gene expression detection and quantification. *Nucleic Acids Research*.

[45] Espuny-Camacho I., Michelsen K. A., Gall D. (2013). Pyramidal neurons derived from human pluripotent stem cells integrate efficiently into mouse brain circuits in vivo. *Neuron*.

[46] Giachino C., Basak O., Taylor V. (2009). Isolation and manipulation of mammalian neural stem cells in vitro. *Stem Cells in Regenerative Medicine*.

[47] Bez A., Corsini E., Curti D. (2003). Neurosphere and neurosphere-forming cells: morphological and ultrastructural characterization. *Brain Research*.

[48] Xiong F., Gao H., Zhen Y. (2011). Optimal time for passaging neurospheres based on primary neural stem cell cultures. *Cytotechnology*.

[49] Zhang X., Huang C. T., Chen J. (2010). Pax6 is a human neuroectoderm cell fate determinant. *Cell Stem Cell*.

[50] Ellis P., Fagan B. M., Magness S. T. (2004). SOX2, a persistent marker for multipotential neural stem cells derived from embryonic stem cells, the embryo or the adult. *Developmental Neuroscience*.

[51] Lendahl U., Zimmerman L. B., McKay R. D. G. (1990). CNS stem cells express a new class of intermediate filament protein. *Cell*.

[52] Gilyarov A. V. (2008). Nestin in central nervous system cells. *Neuroscience and Behavioral Physiology*.

[53] Okabe S., Forsberg-Nilsson K., Spiro A. C., Segal M., McKay R. D. G. (1996). Development of neuronal precursor cells and functional postmitotic neurons from embryonic stem cells in vitro. *Mechanisms of Development*.

[54] Shi Y., Kirwan P., Livesey F. J. (2012). Directed differentiation of human pluripotent stem cells to cerebral cortex neurons and neural networks. *Nature Protocols*.

[55] Johnson M. B., Wang P. P., Atabay K. D. (2015). Single-cell analysis reveals transcriptional heterogeneity of neural progenitors in human cortex. *Nature Neuroscience*.

[56] Gorris R., Fischer J., Erwes K. L. (2015). Pluripotent stem cell-derived radial glia-like cells as stable intermediate for efficient generation of human oligodendrocytes. *Glia*.

[57] Narayanan G., Poonepalli A., Chen J. (2012). Single-cell mRNA profiling identifies progenitor subclasses in neurospheres. *Stem Cells and Development*.

[58] Hutton S. R., Pevny L. H. (2011). SOX2 expression levels distinguish between neural progenitor populations of the developing dorsal telencephalon. *Developmental Biology*.

[59] Yuan T., Liao W., Feng N.-H. (2013). Human induced pluripotent stem cell-derived neural stem cells survive, migrate, differentiate, and improve neurologic function in a rat model of middle cerebral artery occlusion. *Stem Cell Research & Therapy*.

[60] Gómez-López S., Wiskow O., Favaro R. (2011). Sox2 and Pax6 maintain the proliferative and developmental potential of gliogenic neural stem cells in vitro. *Glia*.

